# Health Impact of Drinking Water Quality on the Occurrence of Osteoporosis in Gaza Strip, Palestine

**DOI:** 10.4314/ejhs.v33i5.14

**Published:** 2023-09

**Authors:** Mazen AbuQamar, Mohammed I Tabash, Adnan Aish, Abd-Rabo Abu Hasheesh, Fulla Sharaf

**Affiliations:** 1 Department of Nursing, Faculty of Applied Medical Sciences, Al-Azhar University, Gaza; 2 Department of Laboratory Medicine, Faculty of Applied Medical Sciences, Al-Azhar University, Gaza, Palestine; 3 Department of Earth Sciences, Faculty of Science, Al Azhar University, Gaza, Palestine; 4 Palestinian-German Diagnostic Center in Gaza City; 5 Ministry of Health

**Keywords:** Water quality, osteoporosis, Gaza Strip

## Abstract

**Background:**

Improving water supply quality could be essential for disease prevention strategy that promotes human health. The study aims to investigate the relationship between drinking water quality and the occurrence of osteoporosis in Gaza Strip.

**Methods:**

A case-control study design was used, and a multistage sampling method was employed at the main orthopedic clinic. Participants included 200 individuals diagnosed with osteoporosis and 200 without osteoporosis. All subjects underwent a DEXA scan, and drinking water samples for chemical analysis were done. A structured face-to-face interview was conducted Statistical analysis was performed using SPSS 26, and both descriptive and inferential statistics (chi-square and binary logistic regression) were used.

**Results:**

Factors such as the source of drinking and cooking water, lifestyle, and socioeconomic status were found important in the occurrence of osteoporosis. The source of drinking, cooking water, lifestyle, and socioeconomic played a significant impact in the development of osteoporosis. Bivariate analysis revealed that a number of factors, including female gender, low physical activity, older age (41–50), inadequate education, drinking and cooking water source, and older age (41–50), had a statistically significant association with osteoporosis. With the exception of Mg (>PH =.105, Ca =.102, Mg =.046), the chemical water quality parameter had an impact on the occurrence of osteoporosis but did not achieve a significant difference. Osteoporosis was less likely to occur in people who were obese. Age, obesity, and the lack of magnesium in drinking water were independent predictors of osteoporosis.

**Conclusions:**

The study has identified the need for preventive measures to improve drinking water quality to reduce the incidence of various health conditions, including osteoporosis.

## Introduction

Osteoporosis is a condition in which the bones become weak and brittle, making them more susceptible to fractures. It is more common in developing countries, where the prevalence is 22.1%, compared to developed countries, where the prevalence is 14.5%. People with osteoporosis may experience bone pain and are at an increased risk of fractures (1).

Calcium is an essential mineral for maintaining healthy bones. It helps to form and strengthen bones. Calcium-rich mineral waters can also be a good source of calcium, as the bioavailability of calcium from these sources for preventing and treating osteoporosis and maintaining overall bone health (2). Calcium is essential for the body's normal functioning and proper development and maintenance of strong bones (3). There is an inverse association between calcium intake from drinking water and men's risk of hip fractures (4). Magnesium is another mineral that is important for bone health. It plays a role in the metabolism of calcium and helps regulate the calcium levels in the body. Higher magnesium levels in drinking water may be associated with a lower risk of osteoporotic fractures (2).

Several small private reverse osmosis desalination plants provide drinking water to communities in the Gaza Strip, and these plants are constructed and operated in all governorates (2). The study aims to investigate the relationship between the calcium and magnesium concentrations in drinking water and osteoporosis in patients from 20-50 years old in the Gaza Strip. There is currently a lack of studies on this topic, and the results of this study can be used as baseline data for future research.

## Methods

**Study design**: Case-control design was used. The study was conducted at the Palestinian-German Diagnostic Center in Gaza City and obtained approval from the ethical committee of the Ministry of Health in the Gaza Strip (Helsinki Committee) before starting the research. The study focused on adult clients who attended the center and had a DEXA scan during the research period (2018-2019). DEXA stands for dual-energy X-ray absorptiometry, and it is a type of imaging test that is used to measure bone density. It is often used to diagnose osteoporosis and to monitor the effectiveness of treatments for this condition (3).

**Data collection**: The study used a proportional stratified consecutive sampling that was used at the main orthopedic clinic, including a DEXA scan. The sample size was calculated to be 189 cases using Epi info Seven software, and 11 cases were added to compensate for potential withdrawals. This sampling method was used to study a specific population and to ensure that the sample is representative of the larger population. A multistage sampling method can select a sample representative of the population of interest and provide reliable and accurate results. A Case is a group known to have osteoporosis (DEXA t-score ≤-2.5)

Adult males or females diagnosed with osteoporosis by DEXA and aged 20 - 50 years old were recruited into the study as a cases. Control included the same-age adult males or females who were diagnosed with osteoporosis by DEXA (DEXA t-score above -1). The exclusion criteria (for cases and controls) were the subjects who suffered from chronic diseases such as cancer, asthma, and Crohn's disease such as post menopause women, irregular menstruation, estrogen disorders, patients with liver diseases or renal insufficiency, in addition to patients with hemophilia.

A pilot study was conducted with a small sample of 20 participants. This pilot study allowed us to test the tools and procedures used and make any necessary adjustments. The data collection for this study included DEXA evaluations and interview questionnaires and structured face-to-face interviews with 200 cases of osteoporosis and 200 controls without the condition. Additionally, water samples were collected from participants and analyzed for PH, TDS, Ca, Mg, F, K, and Na at the Sabha Clinical Center (MOH) using standard methods (4).

**Data analysis**: SPSS 25 software was used to perform statistical analysis on a dataset to identify the relationship between risk factors and osteoporosis. Descriptive statistics was used to summarize the data, including frequency, percentage, and cross-tabulation. Inferential statistics, including chi-square and logistic regression, were used to assess the association between the variables. The logistic regression model was used specifically for binary outcomes, such as the presence or absence of osteoporosis, and dummy variables were used to encode the independent variables for easy interpretation. The analysis results were considered significant if the P-value was less than 0.05 and the confidence interval was 95%.

**Ethical Considerations:** Approval was received from all participants, including the ethical committee of the Ministry of Health in the Gaza Strip (Helsinki Committee) prior to the start of the study.

## Results

The study revealed that 58% of the participants were females, with 64% being cases and 52% being controls. There was a significant association (P < 0.049) between osteoporosis and gender. The mean age of the participants was 42.04 (± 9.28) years for cases and 41.00 (± 7.97) years for controls, with the age range being 23 to 50 years and being divided into three groups. The highest percentage of cases (68%) was found in the age group 41-50 years, followed by the age group 31-40 years, while the lowest percentage was recorded in the age group 23-30 years. There was also a significant association (P < 0.008) between osteoporosis and age. In terms of residency, 59% of cases and 65.5% of controls lived in the city, 25.5% of cases and 24.5% of controls lived in a camp, and 15.5% of cases and 10% lived in a village. There was no significant association (P = 0.397) between osteoporosis and residency type.

Regarding marital status, the married participants were 81% among cases compared to 77.5% among controls. The risk of osteoporosis was 1.26 times higher among married participants than non-married participants. However, there was no significant association (P = 0.634) between osteoporosis and marital status. Among female participants, 75% of cases and 73% of controls were married. Table 1 illustrates that 57% of cases Vs. 48.5% of controls are secondary or less (low educational level). The results reflected a statistically significant association between osteoporosis and low educational level (P = 0.044).

**Table 1:** Demographic and socioeconomic characteristics of the study population

Item	Cases n=200	%	Controls n=200	%	*χ* ^2^	OR (CI)	P-value
**Gender**							
Male	72	36	96	48	2.63	1.64 (1.089-3.527)	0.049*
Female	128	64	104	52			
**Age**							
23-30	26	13	56	28	3.85	2.26 (1.281-4.183)	0.008*
31-40	38	19	46	23			
41-50	136	68	98	49			
**Residency**							
City	118	59	131	65.5	1.86	0.57 (0.037-1.526)	0.397
Camp	51	25.5	49	24.5			
Village	31	15.5	20	10			
**Marital status**							
Single	38	19	45	22.5	1.26	1.16(0.023-1.062)	0.634
Married	162	81	155	77.5			
**Female marital status**							
Married	150	75	146	73	1.39	1.01 (0.152-1.271)	0.635
Non-married	50	25	54	27			
**Educational level**							
Secondary or less	114	57	97	48.5	2.15	1.77 (1.183-3.217)	0.044*
Diploma and above	86	43	103	51.5			
**Occupational status**							
Employed	55	27.5	63	31.5	1.67	1.31 (0.092-1.942)	0.273
Unemployed	145	72.5	137	68.5			
**Monthly income in NIS**							
≤ 1800	152	76	139	69.5	1.87	1.43 (.892-1.982)	0.105
> 1800	48	24	61	30.5			

P-value: P- value of chi-square test (χ^2^), P-value > 0.05: Statistically insignificant

* P-value < 0.05 (Statistically Significant), OR: odds ratio

Regarding the occupation status, the unemployed participants were 72.5% among the cases compared to 68.5% among the controls. The finding also showed that unemployed participants were 1.31 times more likely to have osteoporosis than employed participants. This reflected not a statistically significant association between unemployment and the occurrence of osteoporosis (P = 0.273). The study reveals that 75% of the cases Vs. 73% of the controls had a monthly income of less than 1800 New Israeli Shekel (NIS) and no statistically significant association between low monthly income and osteoporosis development (P = 0.635). The risk for osteoporosis concerning physical activity in the study population was classified into two groups. About 63.5% of the case Vs. 51% of controls were physically active, while 36.5% of the cases Vs. 49% of the controls were walking 1-2 miles daily. There was a significant association (P = 0.032) between osteoporosis and low physical activity.

The findings revealed that 52.8% of male cases were smokers for more than five years compared to 46.9% of controls. The risk of having osteoporosis among smokers was 1.78 times higher than among non-smoker participants. This means an increased risk of osteoporosis among people exposed to smoking, and there is no statistically significant association between current smoking and osteoporosis (P = 0.051).

Standard weight status categories associated with BMI ranges for adults are underweight when BMI < 18.5, normal when BMI ranges between 18.5-24.9, overweight when BMI ranges between 25-29.9, and obese when BMI ranges≥ 30 (5,6). Table 2 reveals that participants were classified into two groups according to BMI. The results showed that 20.5% of the cases Vs. and 34.5% of the controls were obese. There was a relationship between obesity and the development of osteoporosis (P = 0.014).

**Table 2:** Lifestyle characteristics of the study population

Item	Cases n=200	%	Controls n=200	%	χ^2^	OR (CI)	P-value
**Physical activity: Walk 1-2 mile**							
Yes	73	36.5	98	49	3.455	1.89 (1.418-3.021)	0.032*
No	127	63.5	102	51			
**Male: Current smoking more than 5 years**							
No	34	47.2	51	53.1	2.264	1.78 (0.923-2.514)	0.051
Yes	38	52.8	45	46.9			
**BMI**							
Underweight-normal	159	79.5	131	65.5	2.867	2.02(1.947-4.182)	0.014*
Overweight - obese	41	20.5	69	34.5			

P-value: P- value of chi-square test (χ^2^), P-value > 0.05: Statistically insignificant

* P-value < 0.05 (Statistically Significant), OR: odds ratio

Table 3 shows that 92% of cases and 83% of controls drank desalinated water. The risk of osteoporosis was 2.92 times higher among participants who drank desalinated water than those who drank municipal water. There was a statistically significant association (P = 0.009) between drinking desalinated water and osteoporosis. The results indicate that 89% of cases and 79.5% of controls used desalinated water for cooking. The risk of osteoporosis was twice as high among participants who used desalinated water for cooking as those who did not. There was a statistically significant association (P = 0.012) between using desalinated water for cooking and the occurrence of osteoporosis.

**Table 3:** Environmental factors of the study population

Item	Cases n=200	%	Controls n=200	%	χ^2^	OR	P-value
**Source of drinking water**							
Municipals water	16	8	34	17.0	3.795	2.92	0.009*
Desalinated water	184	92	166	83.0			
**Cooking water**							
Municipals water	22	11	41	20.5	3.217	2.85	0.012*
Desalinated water	178	89	159	79.5			

P-value: P- value of chi-square test (χ^2^), P-value > 0.05: Statistically insignificant

* P-value < 0.05 (Statistically Significant), OR: odds ratio.

**Water quality analysis**: The results of the water quality analysis showed that the concentration of calcium, magnesium, and fluoride in the water was below recommended levels according to WHO guidelines (Table 4). Based on the results, there is a trend in the levels of calcium (Ca) and magnesium (Mg) in relation to osteoporosis, but the analysis of the water samples did not show a statistically significant difference between the levels of these minerals in the case and control groups (Table 5).

**Table 4:** Comparison of physicochemical water quality with WHO drinking water guidelines

Parameters	Minimum	Maximum	Median	Average	Standard deviation	WHO
**pH**	4.18	8.53	6.56	6.51	0.79	6.5-8.5
**TDS(mg/l)**	10.00	792.00	99.00	121.21	32.41	1000
**Ca 2+ (mg/l)**	1.00	1394.00	2.00	26.93	97.96	100
**Mg 2+ (mg/l)**	0.00	49.00	2.00	4.01	4.93	60
**F (mg/l)**	0.00	24.00	0.14	3.91	3.37	1.5
**K + (mg/l)**	0.14	1.10	0.90	0.25	0.09	5
**Na + (mg/l)**	0.00	29.00	19.00	1.50	2.09	200

*WHO World Health Organization

**Table 5:** Comparison of drinking water quality of the study population

Items	Case/Control	Mean	Std. Deviation	t	Sig.
**PH**	Control	6.49	0.47	-1.92	.105
	Case	6.62	0.51		
**TDS**	Control	102.50	36.21	-2.28	.094
	Case	131.20	46.32		
**Ca**	Control	4.58	5.03	1.83	.102
	Case	3.15	2.59		
**Mg**	Control	4.48	3.10	2.94	.046
	Case	3.01	5.20		
**F**	Control	0.12	0.10	.19	.827
	Case	0.16	0.10		
**K**	Control	0.64	0.80	-.418	.628
	Case	0.69	0.90		
**Na**	Control	27.94	11.36	-1.04	.374
	Case	30.12	13.31		

**Logistic regression**: Table 6 used a model to predict the association between various risk factors and osteoporosis in adults in the Gaza Strip. When the variables were tested without adjusting for confounding factors, there was a statistically significant association between the occurrence of osteoporosis and several factors, including gender, age, education, physical activity, the source of drinking water, the source of cooking water, and Mg. However, after adjusting for these factors in a logistic regression analysis, the significant association between osteoporosis and several factors (gender, education, physical activity, source of drinking water, and source of cooking water) was no longer present (P = 0.053, 0.283, 0.094, 0.096, respectively). Calcium still showed a trend toward being important in the occurrence of osteoporosis. The significant association between osteoporosis and gender, BMI, and Mg remained (P = 0.053, 0.047, 0.049, respectively).

**Table 6:** Logistic regression of risk factors of osteoporosis among the study population

Variables	Wald	P-value	P* value	OR*
**Gender: female**	1.98	.049	0.053	1.68
**Age:41-50 year**	1.68	.008	.049	2.13
**Education: less than secondary**	1.19	.044	.283	1.16
**Income less than 1800 NIS**	1.03	.103	.451	1.08
**Male smoker more than** 5 **year**	1.31	.051	.312	1.17
**Physical activity: < 1 mile daily**	2.94	0.032	.094	1.94
**BMI: not obese or overweight**	2.44	.014	.047	2.14
**Source of cooking water: desalinated water**	1.85	.009	.096	1.42
**Source of drinking water: desalinated water**	1.79	.012	.126	1.47
**>PH**	1.34	.105	.312	1.12
**<Ca**	1.42	.102	.325	1.24

**<Mg**	3.42	0.046	.049	1.82


*P*-value: *P*-value of logistic regression, P-value > 0.05: Statistically insignificant

* *P*-value < 0.05(Statistically significant), OR: adjusted odds ratio.

**Interaction effects (combination effect)**: According to the data, there was a significant association between the occurrence of osteoporosis and a combination of female gender and older age (41-50) (p-value = .013 and OR = 3.45). Female gender also had a significant effect on osteoporosis when combined with low levels of Mg in the household water supply (P-value = 0.009 and OR = 3.67). Similarly, there was a significant association between the occurrence of osteoporosis, female gender, and low physical activity (walking less than 1 mile daily) (P-value = 0.013 and OR = 2.24). Physical activity is known to increase bone accretion during growth. This can help to reduce the risk of osteoporosis (7). The study found that the prevalence of osteoporosis was much higher among females than males (86.5% vs. 13.5%).

## Discussion

This study highlighted the impact of the source of drinking and cooking water, lifestyle, and socioeconomic status on the occurrence of osteoporosis. The study aimed to investigate the relationship between drinking water quality and osteoporosis in the Gaza Strip. In developing countries, the per capita drinking water cannot satisfy 50% of the minimum requirement (8).

Unsafe drinking water is associated with high morbidity and mortality from related diseases (9). The scientific literature has supported our findings when it has linked declining in the quality of drinking water associated with the occurrence of osteoporosis. A poor socioeconomic background was significantly associated with osteoporosis occurrence, and there were disparities in socioeconomic status between the cases and controls. Previous studies have consistently shown that women are at a higher risk of developing osteoporosis due to their smaller bones and lower overall bone mass, which agrees with our findings (1,4). After menopause, women experience a more rapid loss of bone mass and tend to live longer. On the other hand, men typically lose less bone mass on average (10–13). Prolonged breastfeeding has been suggested to have a potential long-term effect on bone mineral density (BMD) and may increase the risk of osteoporosis in later life (14,15).

Many recent research studies supported our findings when they showed a variation in the incidence of osteoporosis between urban and rural inhabitants. These studies also found that overall fracture rates were higher among county residents in the city center than in rural areas (16,17). Filip and Zagorski's (2001) research showed that the mean bone mineral density (BMD) between urban and rural populations did not significantly differ. Therefore, it is still unclear if there is a direct correlation between where one lives and the development of osteoporosis. More research is needed to understand the relationship between osteoporosis and the living environment (16,18).

In this study, educational level has a crucial impact on the incidence of osteoporosis. According to a study by Etemadifar et al. (2013), women with higher education tend to have a greater understanding of osteoporosis compared to those with less education and health education, and there is a notable impact on providing a safe water supply (19,20). Only a few studies' findings agreed with this (21,22). Other studies have found that high income and food security are strongly linked to a lower risk of osteoporosis and fractures (23,24).

This means an increased risk of osteoporosis among people exposed to smoking, and there is no statistically significant association between current smoking and osteoporosis (P = 0.051). Smoking has been shown to negatively affect bone health by impacting 1, 25-dihydroxy vitamin D and estrogen levels. It also increases the likelihood of menopausal changes in women, which are associated with an increased risk of osteoporosis (25,26). However, it is known that quitting smoking can partially reverse the bone loss caused by smoking and has numerous positive effects on a person's overall health (27).

Obesity is a protective factor against osteoporosis, as evidenced by the negative relationship between general obesity and femoral neck osteoporosis (28). Being overweight (BMI > 25-29.9) was protective or neutral for bone mineral density (BMD); however, obesity (BMI > 30) was linked to poor bone mass, which is consistent with an osteoporosis diagnosis (29).

The results of the water quality analysis showed that the concentration of calcium, magnesium, and fluoride in the water was below recommended levels according to WHO guidelines (30). The process of desalination, which involves removing salts and minerals from water, can negatively impact health. Table 4 Compares the physicochemical water quality with WHO drinking water guidelines.

Based on the results, there is a trend in the levels of calcium (Ca) and magnesium (Mg) in relation to osteoporosis, but the analysis of the water samples did not show a statistically significant difference between the levels of these minerals in the case and control groups. This could be due to the absence of Ca and Mg in both the case and control groups. However, the results also suggest that socioeconomic and lifestyle factors such as income, education, and smoking may contribute to an increased incidence of osteoporosis, as shown in Table 5. In a study by Ayele et al., the authors described low vitamin D (Vit D) in 96% of 25 Ethiopian multiple scleroses (MS) patients, with a mean age of 35.8 years (1,13). Among them, 50% of the patients had severe hypovitaminosis D (31). A Norwegian study found an inverse association between distal forearm bone mineral density and the frequency of consumption of soft drinks and fruits and vegetables. Since BMD has been the focus of the majority of studies on magnesium and bone, it is yet unknown how magnesium may affect osteoporotic fractures. Research shows that too low and too high magnesium consumption may be hazardous to bone health, even though magnesium deficiency is more prevalent than excess (32,33).

Similarly, there was a significant association between the occurrence of osteoporosis, female gender, and low physical activity (walking less than 1 mile daily) (P-value = 0.013 and OR = 2.24). Physical activity is known to increase bone accretion during growth. This can help to reduce the risk of osteoporosis (7). The study found that the prevalence of osteoporosis was much higher among females than males (86.5% Vs. 13.5%). This result is consistent with many other studies that reported that women are at a higher risk of developing osteoporosis than men. Women generally have smaller bones and lower total bone mass, which puts them at a higher risk of osteoporosis (1). It is also important to note that women tend to lose bone more quickly after menopause and typically live longer than men, which further increases their risk of developing osteoporosis. While osteoporosis is less common in men, it is still a significant problem. Men have a slower bone loss rate than women, but a recent study showed that osteoporotic or fragility fractures still affect one in two women and one in five men over 50 years old. These fractures can lead to significant morbidity, increased mortality, and a decreased quality of life (10,34).

To our knowledge, no previous studies have shown the relationship between the quality of drinking water and different socioeconomic factors with the occurrence of osteoporosis at the same time. This especially holds for studies made in the Gaza Strip. Our study is also original in its design because it models the intra-relationship of independent variables and adjusts for other confounders that have a significant association with osteoporosis occurrence.

The study indicated that certain minerals in the water, lifestyle factors, and socioeconomic factors may contribute to the development of osteoporosis. Many of these factors have also been linked to the development of other chronic diseases. The study also found that osteoporosis is more prevalent among females than males, and the prevalence of osteoporosis increases with age in both sexes. In addition, having multiple pregnancies, low educational level, and unemployment were identified as independent risk factors for osteoporosis. The study revealed that increasing the concentration of magnesium in drinking water may be an effective measure for protecting against osteoporotic fractures in the population. Ensuring the water is free of harmful microorganisms may also benefit bone health.

Based on these findings, healthcare planners, water authorities, and policymakers need to implement corrective strategies to improve awareness about the relationship between the source of drinking water, knowledge, attitude, and practice, and the occurrence of osteoporosis in the Gaza Strip.
